# Fatty acid synthesis and NLRP3-inflammasome

**DOI:** 10.18632/oncotarget.4781

**Published:** 2015-07-03

**Authors:** Jong-Seok Moon, Kiichi Nakahira, Augustine M.K. Choi

**Affiliations:** Joan and Sanford I. Weill Department of Medicine, Weill Cornell Medical Center, New York, NY, USA

**Keywords:** Immunology and Microbiology Section, Immune response, Immunity, fatty acid synthesis, NLRP3 inflammasome

Fatty acid synthesis is an important process of the lipogenesis [1]. Fatty acid synthesis is tightly regulated by key enzymes including fatty acid synthase (FASN) [1]. FASN and lipogenesis related enzymes are upregulated in various cancer cells and associated with oncogenic progression [1]. FASN is being an indicator of poor prognosis and a chemotherapeutic target in breast cancers [1]. Fatty acid synthesis has been implicated in immune response such as the differentiation of B lymphocytes and human monocytes [2, 3]. Recently, we demonstrated that upregulation of FASN-dependent fatty acid synthesis regulates inflammation through NLRP3 inflammasome activation in macrophages [4].

We have described a novel molecular mechanism that fatty acid synthesis under the regulation of FASN is critical for NLRP3-mediated caspase-1 activation. Genetic and pharmacologic inhibition of FASN suppressed NLRP3-mediated caspase-1 activation in macrophages, as well as IL-1β and IL-18 production *in vivo* [4]. Our study revealed that FASN-dependent lipid synthesis acts as an upstream signal for *NLRP3* gene expression [4]. Inhibition of fatty acid synthesis by FASN knockdown or C75, a potent FASN inhibitors, suppressed the NLRP3 inflammasome activation in macrophages [4].

FASN is a multifunctional metabolic enzyme that catalyzes the terminal steps in the synthesis of long-chain saturated fatty acids [1]. The production of fatty acids supports membrane synthesis in proliferating cells [1]. Furthermore, FASN expression and activity is critical for AKT activation which promotes cell survival and enhances tumor cell growth and invasiveness [1]. We demonstrated that FASN regulates *NLRP3* gene expression *via* AKT activation in macrophages [4]. The activation of AKT in response to lipopolysaccharide (LPS) stimulation is suppressed by FASN inhibition [4]. Consistently, inhibition of AKT by BX795, a selective AKT inhibitor, reduced *NLRP3* gene expression [4]. Our results suggest that FASN regulates the activation of AKT signaling pathway in macrophages.

Activation of FASN can promote glucose-dependent fatty acid synthesis, illustrating that glucose utilization is regulated by lipogenesis [1]. Because the metabolites from glycolysis are the main carbon sources for lipid synthesis, glycolysis can be regulated along with fatty acid synthesis. It has been shown that glycolysis is linked to changes in lipogenesis required for cell proliferation in androgen-dependent prostate cancer cells [5]. Similarly, we identified that increased glycolytic phenotype is linked to activation of FASN-dependent fatty acid synthesis during inflammation *in vitro* and *in vivo* [4].

Since FASN has a critical role in inflammation and cancer progression [1–3], several studies have attempted to identify an upstream regulator of FASN as a molecular target for anti-inflammatory or cancer treatment. Previous study has shown that the expression of FASN is increased by human epithelial growth factor receptor-2 (HER2) overexpression, and that the activation of the mammalian target of rapamycin (mTOR) by HER2 plays an important role in their overexpression through the selective translational activation in breast cancer cells [6]. Similarly, we identified that UCP2 regulates fatty acid synthesis during inflammation in macrophages. Deficiency of UCP2 reduced the expression of FASN and triglyceride synthesis in response to LPS in macrophages [4]. Our results demonstrated that UCP2 plays an upstream regulator of FASN-dependent lipid synthesis in macrophages.

Several types of inflammation by different causes and mechanisms can promote cancer development [1]. Recent study has reported that 5-FU and Gemcitabine mediated NLRP3 inflammasome activation represent a new mechanism of resistance to chemotherapy treatment in cancer [7]. Chronic inflammation and fatty acid metabolism could be linked as critical events in carcinogenesis and tumor progression. We identified that FASN-dependent fatty acid synthesis regulates the NLRP3 inflammasome activation in macrophages [4]. Our results suggest that FASN-dependent fatty acid synthesis might be a target for regulating of by chemotherapy treatment in cancer. Collectively, we suggest that FASN could be a critical regulator of the NLRP3 inflammsome activation in cancer.
